# Exploring the Relationship Between Childhood Maltreatment and Addiction: A Review of the Neurocognitive Evidence

**DOI:** 10.1007/s40429-015-0073-8

**Published:** 2015-10-01

**Authors:** Vanessa B. Puetz, Eamon McCrory

**Affiliations:** Division of Psychology and Language Sciences, University College London, 26 Bedford Way, London, UK

**Keywords:** Childhood maltreatment, Addiction, Substance use disorders (SUDs), Latent vulnerability, Reward processing, Amygdala, Prefrontal cortex

## Abstract

Childhood maltreatment has been shown to increase the risk of a range of psychiatric disorders including substance use disorders (SUDs) and is associated with the onset, course and severity of illness. We review the evidence for alterations in brain structure and neurocognitive processing in individuals who have experienced childhood maltreatment, focusing specifically on changes related to reward processing, executive functioning and affect processing. Changes in these neurocognitive systems have been documented in adults presenting with SUDs, who are typically characterized by heightened subcortico-striatal responses to salient stimuli and impairments in fronto-cingulate regulation. Maltreatment-specific effects in these processing domains may account for the particularly severe clinical presentation of SUDs in adults with histories of maltreatment in childhood. The findings are considered in relation to the theory of latent vulnerability, which contends that alterations in these neurocognitive systems may reflect calibration to early risk environments that in turn increases the risk of developing of SUDs later in life.

## Introduction

Childhood maltreatment, including physical and emotional neglect, has been identified as a major risk factor for a variety of negative outcomes spanning the domain of mental and physical health as well as economic attainment [1]. Specifically, childhood maltreatment has been shown to increase the risk for a range of psychiatric disorders throughout the lifespan [2], notably affective disorders such as depression and anxiety [3], conduct disorder [4] and substance abuse [5••].

The theory of latent vulnerability has been proposed to account for the link between maltreatment and psychiatric disorder [6••]. According to this account, childhood maltreatment results in alterations at multiple levels of neurobiological and cognitive functioning, reflecting systems-level calibrations in response to early risk environments. While such changes may have short-term benefits, they can serve to embed heightened vulnerability to psychiatric disorder in adolescence or adulthood following exposure to future stressors. This systems-level approach implies that stress-related changes in certain candidate neurocognitive systems are theoretically measurable in childhood and constitute markers for future psychiatric risk.

This notion of latent vulnerability provides a helpful framework within which to study substance use disorders (SUDs), one of the most robust psychiatric disorders associated with childhood maltreatment [5••, 7]. SUDs are estimated to present in nearly half of all adults who experienced maltreatment in childhood [8], typically manifesting earlier [9] and with greater severity in these individuals compared to those with SUDs who had not experienced maltreatment [10, 11]. In addition, previous experiences of childhood maltreatment appear to significantly affect the course of treatment, as they are associated with greater risk of dropout and comorbidity with other psychiatric disorders and shorter time to relapse [10, 12, 13••]. While neurobiological studies of adults presenting with SUDs suggest that the experience of childhood maltreatment may alter brain structure and function in ways that embed latent vulnerability, surprisingly little is still known about the possible mechanisms that may link maltreatment experience and the emergence of substance use problems in adulthood. Here, we review the evidence for three candidate neurobiological systems that may be shaped by early maltreatment experience in ways that heighten vulnerability of SUDs in adulthood: (1) reward processing, (2) executive functioning and (3) threat processing.

### Reward Processing

The ability to seek out stimuli that have incentive salience such as appetitive cues (e.g. food and sex) or social rewards (e.g. smiles and praise) governs most motivational behaviour in animals and humans. The neural systems underlying reward processing and motivational behaviour include the ventral striatum (comprising the caudate nucleus, globus pallidus and the nucleus accumbens), the anterior cingulate cortex (ACC), the medial prefrontal cortex (including the orbitofrontal cortex (OFC)) and the amygdala, jointly forming the mesocorticolimbic reward circuitry. Alterations in the reward system can interfere with an individual’s ability to goal-direct their behaviour and decision-making, and reduced or altered reward processing is a hallmark feature of maltreatment-related psychopathology such as major depression, PTSD and especially substance abuse [14–16]. Indeed, neural and behavioural adaptations of the reward systems are cardinal features of substance abuse in particular and are associated with altered conditioned stimulus reward associations leading to heightened incentive salience of drug-related cues and blunted responses to natural rewards [17].

There is an established relationship between stress and altered reward sensitivity in the animal literature showing that chronically stressed animals show a reduced behavioural motivation to obtain appetitive food cues, suggesting hyposensitivity to reward following stress [18]. The few studies to date that have investigated sensitivity to rewards in children and adolescents exposed to childhood maltreatment suggest that maltreatment-related stress might also alter reward processing in humans at both neural and behavioural levels. In the first study to investigate reward processing in children with maltreatment experiences, Guyer and colleagues found reduced sensitivity to monetary rewards in maltreated children [19]. The authors showed that these children responded faster than their non-maltreated peers irrespective of reward value (high vs. low) but did not accelerate their response speed with increasing likelihood of winning a monetary reward. By contrast, control children responded faster with increasing chance of winning. This relative insensitivity to changing reward values suggests heightened impulsivity as well as diminished goal-directed behaviour and blunted response towards (monetary) rewards in children with a history of maltreatment. However, the study also showed that maltreated children who additionally met the criteria for depression favoured safe choices with smaller gains over riskier choices, suggesting that affective disorders may mediate differential patterns of motivational behaviour in this population. Similar findings have been obtained in a study investigating reward processing in young adults with a history of childhood maltreatment using functional magnetic resonance imaging (fMRI) [20]. Compared to young adults without a history of childhood maltreatment, adults who experienced childhood maltreatment subjectively rated cues signalling a potential reward (vs. loss) as less positive, suggesting that alterations occur at the earliest stage of reward processing, i.e. the anticipation of reward cues. These behavioural changes were associated with reduced activation in the globus pallidus to cues signalling gains but similar activation to the gains themselves. Comparable findings of reduced striatal activation during the anticipation of reward were also obtained in a study of adolescents who were adopted from Romanian institutions in a monetary reward task [21]. Complimenting these functional investigations, studies investigating structural brain architecture have found structural brain alterations in the caudate nuclei [22], the ACC [23] and reduced white matter integrity in fronto-striatal pathways [24] in individuals who have experienced maltreatment, patterns which are remarkably consistent with alterations in neural structure shown to characterize individuals with SUDs [25].

Collectively, these findings suggest that stress-related alterations in the subjective and neural processing of reward anticipation may follow experiences of childhood maltreatment, with important implications for decision-making and the ability to initiate goal-directed actions. While these changes may potentiate the development of SUDs later in development, the evidence at the current time is preliminary in nature. Next, we review studies that have explicitly investigated reward function in individuals presenting with SUDs with and without experiences of childhood maltreatment.

It is now well established that individuals who abuse substances are characterized by altered brain functioning in the reward system, specifically within the fronto-striatal and fronto-limbic pathways, consistent with behavioural evidence of altered motivational behaviour and impulsivity in response to drug-related cues [26]. Specifically, and paralleling findings of blunted responses to rewards in maltreated children, studies with individuals with SUDs have consistently shown blunted activity in response to natural rewards, while brain responses towards drug-related cues are amplified [17]. However, only recently have researchers begun to investigate reward processing in individuals with SUDs and a history of childhood maltreatment. For example, a recent PET study has shown that the number of traumatic events that adults had experienced was positively associated with a higher ventral striatal dopamine response to amphetamine in adults, suggesting that childhood maltreatment may exert a dose-dependent influence, such that longer periods of adversity increase neural sensitivity to psychostimulants exponentially [27].

The role of developmental timing of adversity was highlighted in a recent longitudinal study with young adult males by Casement and colleagues [28]. The authors demonstrated that cumulative stressful life events in mid-adolescence were associated with blunted medial prefrontal cortex (mPFC) function in response to monetary reward anticipation and actual reward gain, which was amplified in individuals with more severe stress during adolescence. Importantly, the authors showed that mPFC activity mediated the relationship between stressful life events in adolescence and future alcohol dependence, suggesting that the mPFC might continue to be vulnerable to the effects of stress until late adolescence, consistent with its protracted developmental time course [29, 30].

#### Summary

Three main conclusions may be drawn from the extant literature. First, maltreatment in childhood is associated with behavioural changes as well as alterations in the brain structure and function that are consistent with altered reward processing. Second, maltreatment-related changes in reward processing are remarkably consistent with those reported in adults presenting with SUDs, typically showing blunted responses to natural rewards. Finally, recent studies have reported maltreatment-specific effects on reward processing, which may account for the particularly severe clinical presentation of SUDs in adults with histories of maltreatment in childhood. Important limitations of the studies discussed above include the uniform use of monetary reward cues to index the reward system along with the frequent investigation of all-male samples with a heterogeneous range of early adverse experiences. An important task for future research will be to extend the investigation of reward processing to include female samples as well as other forms of reward, in particular social reward.

### Executive Function and Cognitive Control

Deficits in executive functioning and cognitive control associated with childhood maltreatment are important to consider in the study of SUDs given that impairments in impulse control and behavioural inhibition have consistently been identified as predictors for SUDs [31, 32]. Specifically, deficits in cognitive control and emotion regulation and alterations in the underlying neural substrates associated with these cognitive processes are both known risk factors for the development of SUDs [33••, 34] and also frequently observed in children with maltreatment experience [35, 36••, 37, 38]. To our knowledge, there are no studies to date explicitly investigating the neural correlates of emotion regulation in drug-dependent individuals with histories of childhood maltreatment; however, here, we review the evidence of behavioural and neurocognitive deficits in these domains in the maltreatment literature.

There is now robust evidence that executive functioning deficits characterize children and adolescents who have experienced childhood abuse and neglect, including in relation to working memory, cognitive control and emotion regulation [36••, 37–40]. For example, in a series of studies with adoptees from Romanian orphanages, several researchers demonstrated significant deficits in executive functioning as well as alterations in neural regions thought to underlie executive functioning, i.e. the prefrontal cortex [41, 42].

Structural MRI studies investigating adults who experienced maltreatment as children frequently report smaller prefrontal grey matter volumes, specifically in the dorsolateral and medial prefrontal cortex involved in cognitive control and emotion regulation, respectively [43, 44]. Similarly, community studies of children with documented experiences of childhood maltreatment have reported alterations in prefrontal structures compared to non-maltreated children. Two studies of grey matter volume in community samples of children with documented experiences of maltreatment reported smaller OFC volumes [45, 46] even in the absence of psychiatric disorder; relatedly, reduced integrity of white matter tracts connecting with the OFC have also been reported [47, 48]. It appears that these structural alterations in the OFC may relate to impairments in social functioning [48, 49] as well as impairments in cognitive performance [50], suggesting a link between alterations in brain structure and domains of functioning which may increase risk for psychopathology. Recent surface-based approaches, which are arguably more sensitive to subtle differences in cortical structure, have also reported differences in prefrontal structures [23]. Specifically, reduced cortical thickness has been found in the ACC, superior frontal gyrus as well as the OFC. Importantly, this fine-grained method was the first to reveal structural differences in the ACC in children, potentially representing a precursor to the volumetric ACC differences seen in adults. Considering the extensive connections between the medial prefrontal cortex and subcortical structures such as the amygdala, one possibility is that insufficient prefrontal top-down control over subcortical reactivity may compromise emotional regulation in ways that impairs functioning in social and cognitive domains. Such a hypothesis is consistent with a set of putative causal changes that may link maltreatment experience with later SUDs given the evidence of social and cognitive deficits in adult samples presenting with SUDs [5••, 51].

To date, only a small number of studies have directly investigated the functional neural correlates underlying cognitive and emotional control in individuals with maltreatment experiences. In a study of adult women with childhood experiences of sexual and physical abuse, Cromheeke and colleagues [51] investigated how emotional stimuli influence cognitive control processes using distracting emotional information during a working memory task. Importantly, this study aimed to differentiate between abuse-related childhood stress and non-abuse-related childhood stress by including a control group of woman who reported childhood stress in the absence of abuse (i.e. maltreatment specific) experiences. Women who had experienced abuse showed reduced working memory accuracy for positive relative to neutral emotional stimuli compared with healthy controls and those who had experienced generic stress in childhood. McLaughlin and colleagues [36••] have recently reported the first direct investigation of the neural systems supporting emotion regulation in adolescents with a history of physical and sexual abuse. While maltreated adolescents and non-maltreated peers were found to show similar degrees of amygdala downregulation during cognitive-reappraisal of negative stimuli (vs. passive viewing), maltreated adolescents showed greater activation of prefrontal regions involved in effortful control (superior frontal gyrus, dACC and frontal pole). These differences in emotion regulation are in line with reported differences in cognitive control, as indexed by a Go/No-Go task [37]. Another recent study also implicated reductions in fronto-cingulate pathways in an investigation of the influence of childhood abuse on drug-cue-induced craving in a sample of cocaine-dependent adult men using personalized, script-guided mental imagery to induce drug craving in fMRI [52]. Compared to non-dependent control subjects, the authors found reduced activation in dependent men with a history of abuse in the ACC during stress-induced craving, a structure that has been associated with the resolution of emotional conflict, especially during increased emotional stress [53, 54] In line with this, greater maltreatment severity was associated with reduced activity in key areas involved emotion regulation (mPFC) and greater reported anxiety during the drug-cue-induced craving. The authors suggest that maltreatment-related changes in brain areas associated with emotion regulation in high-stress situations such as the craving state might underlie heightened risk for relapse in drug-dependent individuals who experienced childhood abuse.

#### Summary

Collectively, these preliminary findings point to medium- and long-term influences of maltreatment experiences on emotion regulation and cognitive control of emotion. While research into the structural correlates of executive functioning and emotion regulation have reported robust alterations in prefrontal cortical architecture, functional brain imaging studies of these domains of functioning in maltreated individuals remains relatively sparse. It is noteworthy that the same brain regions associated with emotional and cognitive control in maltreated populations overlap with those identified as potential predictors of SUDs.

### Affect Processing

The study of affect processing in childhood maltreatment has increasing relevance in the understanding of the development of SUDs, since limited, but increasing evidence points towards alterations in affect-related processing including the perception, experience and regulation of emotions in SUDs [55]. Childhood maltreatment has been associated with heightened perceptual salience of negative stimuli, suggesting hypervigilance to threat and differential allocation of attentional resources to threat-related cues. A series of studies have demonstrated that children who experienced childhood maltreatment in the form of physical abuse show preferential attention to angry facial expressions and have greater difficulty to disengage from such threat-related cues [56, 57], suggesting that maltreatment experience shapes affect processing and leads to differential allocation of attentional resources to salient stimuli, even in children as young as 15 months of age [58]. Though limited in number, there are studies showing the same enhanced response to fearful faces in individuals with SUDs [59] and emotional expressions in general [60].

Medial temporal lobe structures such as the amygdala play a key role in the neural processes underlying salience detection, the ability to process negative information and learn stimulus-outcome associations. Findings of structural alterations in amygdala volume in individuals who experienced childhood maltreatment have been mixed, some suggesting no differences in amygdala volume in children with maltreatment-related PTSD relative to non-maltreated peers and others suggesting increased amygdala volumes in previously institutionalized adoptees [61, 62]. These mixed findings may be attributable to the heterogeneity in samples, including differences in the types and lengths of maltreatment experienced, and in the presence of a range of comorbid disorders in the participants included in many of the studies to date. However, somewhat surprisingly given the animal literature, it appears that changes in amygdala volume are not associated with maltreatment experience [63].

By contrast, there is an emerging consensus that childhood maltreatment has a significant impact on amygdala functioning. Several fMRI studies conducted with children who have experienced maltreatment have now reported a heightened amygdala response to angry faces relative to non-maltreated peers [57, 62, 64–66] . One such study by McCrory and colleagues [57] investigated threat processing in maltreated children who were free of comorbid psychopathology. Greater activation in the amygdala and anterior insula was observed in the maltreated group relative to their peers when viewing angry relative to neutral faces. Interestingly, this neural signature is also seen in adult patients with anxiety disorders [53], in individuals with SUDs in a craving state [17] and can be reduced by the anxiolytic effects of alcohol [67]. A follow-up study by McCrory and colleagues, using a masked dot-probe paradigm, investigated the temporal dynamics of the neural response to threatening faces in maltreated children by presenting angry face stimuli for periods of time that are outside conscious awareness (<17 ms, [65]). Results of this study revealed heightened amygdala reactivity in maltreated children relative to non-maltreated peers even during pre-attentive processing of emotional expressions, suggesting alterations at the earliest stages of affect processing. Importantly, several studies have now found a relationship between the extent of amygdala activation to salient stimuli and the onset and timing of the maltreatment experiences, such that younger age of onset and more severe maltreatment experiences were associated with greater amygdala sensitivity [64, 65, 68]. It has been suggested that neurocognitive alterations to threat processing, indexed by heightened amygdala reactivity to salient emotional cues, may represent one possible marker of latent vulnerability. Specifically, greater attunement to threat may represent an adaptive response to an early threatening and unpredictable environment but may incur a long-term cost in increasing vulnerability to psychopathology in more normative environments [6••].

To date, only few studies have investigated affect processing in individuals with SUDs with a history of childhood maltreatment, which is surprising given the anxiolytic potential of some substances to blunt affective reactivity, particularly to threat [67]. Indeed, there is considerable overlap in findings of heightened amygdala reactivity to salient emotional cues in SUDs and maltreatment, thought to specifically affect craving and socio-emotional functioning in SUDs. Recent evidence suggests that automatic maltreatment-related associations even have the potential to activate drug-related associations and elicit feelings of craving [69]. Numerous imaging studies to date have established a relationship between heightened amygdala reactivity and script-guided or video-induced craving, as well as subjective reports of craving in individuals with SUDs, highlighting the importance of the amygdala in conditioned stimulus associations [70–72]. Interestingly, for drug-unrelated, affective stimuli, individuals with SUDs show a pattern of emotional blunting, similar to the blunted response to natural rewards observed in maltreatment and SUDs [73]. However, studies investigating the recognition of facial expressions in SUDs suggest an enhanced response to a range of facial expression including fear [59]. Importantly, in non-dependent healthy controls, alcohol has been shown to attenuate amygdala reactivity to threatening faces, providing a potential mechanism by which the anxiolytic effects of some substances mediate an increased risk for SUDs in maltreated individuals [67].

#### Summary

Findings from fMRI studies investigating affect processing in children with a history of childhood maltreatment and individuals with SUDs have demonstrated both behavioural hypervigilance and heightened amygdala reactivity to threat-related cues. Preliminary evidence from individuals with SUDs and a history of childhood maltreatment points towards similar processes by which conditioned stimulus associations may influence the salience of drug-related stimuli and the degree of the arousal response. Importantly, considering the anxiolytic effects of alcohol on anxiety levels and amygdala reactivity, sustained drug abuse in maltreated individuals could be understood in part as a way of reducing negative affect and heightened arousal, consistent with a self-medication account [73].

## Conclusion

The review of the present literature demonstrates a growing empirical basis to infer a link between the experience of childhood maltreatment and an elevated risk of developing SUDs later in life. Such a putative link may be mediated by alterations in those neurocognitive systems implicated in reward processing, cognitive control and affect processing given that these systems are atypical both in those who have experienced maltreatment in childhood and in those who present with SUDs. It has been suggested that calibration of each of these systems may embed a degree of latent vulnerability, increasing the risk of future psychopathology, including SUDs. We speculate that heightened subcortical and striatal activity to salient cues along with altered capacities for affect regulation and reasoning in maltreated individuals may increase risk for SUDs in three possible ways: first, by altering the balance and strength of responses to drug and natural reward cues, enhancing the former while attenuating the latter; second, by compromising the ability of the individual to regulate their emotional and behavioural responses increasing the likelihood of substance abuse initiation; and third, by increasing the likelihood of self-medication as a result of maltreatment-related increases in internal aversive states combined with impairments in regulatory control.

However, a number of limitations constrain inferences that can be made in this regard. Firstly, most of the evidence reviewed here is cross-sectional making it difficult to disentangle the effects of childhood maltreatment and SUD on brain structure and function. While only longitudinal studies can help inform causal inferences, recent studies have suggested that childhood maltreatment has independent effects on brain structure above and beyond SUD [13••, 74]. For example, a recent study investigated the individual effects of SUD and childhood maltreatment on grey matter development [13••] with whole brain voxel-based morphometry (VBM). In this study, the authors controlled for SUDs and comorbid psychiatric disorders and found reductions in grey matter volume uniquely relating to childhood maltreatment in the hippocampus and parahippocampus that predicted severity of substance use relapse while the SUD-related areas of GMV reduction (e.g. posterior cingulate) did not have predictive value. This supports the notion that childhood maltreatment can contribute independently to alterations in brain structure in SUD and influence the course of illness. Unfortunately, this study also used a cross-sectional design so it remains unclear whether pre-existing individual differences may have influenced the current findings.

In a similar vein, any comprehensive account linking maltreatment and SUDs needs to consider possible genetic factors, given that SUD is highly heritable (for a review of effects of gene expression changes in mesolimbic pathways after childhood maltreatment, see [11]). While it is conceivable that genetic risk plays a major role in the development of SUDs in individuals with maltreatment experiences, it has been argued that it is highly unlikely that these account solely for the increased risk (see, [75]). In addition, gender differences have been demonstrated in the development of SUDs after childhood maltreatment, with childhood maltreatment frequently being more predictive for SUDs in woman than men (see, [76]). Future research, including large sample sizes that allow for the investigation of gender differences, are needed to elucidate if childhood maltreatment interacts with gender to increase risk for psychopathology and especially SUDs. Finally, it is important to keep in mind that not all individuals who experience childhood maltreatment develop psychopathology later in life or engage in substance use behaviours, highlighting the case for a greater understanding of those factors that contribute to resilient outcomes in these individuals.
